# Draft genome of *Kazachstania heterogenica var. weizmannii*, a commensal fungus of the mouse digestive tract

**DOI:** 10.1128/mra.00115-24

**Published:** 2024-03-19

**Authors:** Jarmila Sekeresova Kralova, Lena Fidel, Catalina Donic, Shifra Ben-Dor, Steffen Jung

**Affiliations:** 1 Department of Immunology and Regenerative Biology, Weizmann Institute of Science, Rehovot, Israel; 2 Department of Life Sciences Core Facilities, Weizmann Institute of Science, Rehovot, Israel; University of California Riverside, Riverside, California, USA

**Keywords:** *Kazachstania heterogenica*, genome annotation

## Abstract

*Kazachstania heterogenica* is a member of the *K. telluris* complex, where all members to date are reported to be pathogenic fungi. We have isolated a strain, *K. heterogenica var. weizmannii,* from the gut of mice that seems to be a commensal strain and sequenced its genome.

## ANNOUNCEMENT

*Kazachstania heterogenica* is a fungus in the *Kazachstania telluris* species complex of the family *Saccharomycetaceae*. Other complex members (*K. telluris*, *K. bovina*, *K. slooffiae*, and *K. pintolopesii*) were isolated from various hosts in the context of pathogenicity (1). We isolated a putative commensal fungus from mouse feces while studying the effects of *Candida albicans* infection on immunocompromised mice. Our isolate has not shown signs of pathogenicity (2).

*K. heterogenica var. weizmannii* was cultured from the feces of mice housed in an SPF facility (2) and grown on solid YPD media at 37°C. DNA was isolated from fungal pellets using a DNeasy Blood & Tissue Kit (Qiagen, cat. 69504). Sequencing and hybrid (Nanopore and Illumina) assembly were performed by SeqCenter (Pittsburgh, PA), as follows: Illumina libraries were prepared using an Illumina DNA Prep kit and IDT 10 bp UDI indices, and sequenced on an Illumina NextSeq 2000 producing 2 × 151 bp reads. For all programs mentioned, default parameters were used except where otherwise noted. The data were demultiplexed and adapters were removed using bcl2fastq (v2.20.0.445) (3). Samples were prepared for sequencing using Oxford Nanopore’s “Genomic DNA by Ligation” kit (SQK-LSK109) with no fragmentation or size selection. The samples were run on Nanopore R9 flow cells (R9.4.1) on a MinION. Basecalling was performed with Guppy (version 4.2.2), in high-accuracy mode (Default parameters + effbaf8). Quality control and adapter trimming were performed with Porechop (version 0.2.2_seqan2.1.1) (4). Statistics are shown in Table 1.

**TABLE 1:** Sequencing statistics

Illumina read pairs	5820035
Illumina bases	1,737,163,443 bp
Illumina coverage	121×
Nanopore Raw Read N50	5,450 bp
Nanopore trimmed reads	400,977
Nanopore trimmed bases	951,032,193 bp
Nanopore coverage	66×

Long read assembly with ONT reads was performed with flye (5) (version 2.8). The long read assembly was polished with Pilon (1.23) for one round of base correction (6). Final assembly statistics were recorded with QUAST (5.0.2) (7). The final assembly of 33 contigs has a length of 14.37 Mb, 30.39% GC, and an N50 of 1.03 Mb.

Annotation was performed with the Yeast Gene Annotation Pipeline (YGAP) (8) with the Post-WGD settings, and Companion (9) for Fungi with the reference organism *Candida glabrata* CBS138. The output of both programs was compared with CD-HIT (version 4.8.1) (10) with c = 1 to reduce redundancy, and the remaining genes were combined using YGAP as the base annotation using in-house scripts. The assembly has 5,217 protein-coding genes, 223 tRNAs, and 7 putative long non-coding RNA genes. The genome has 86.9% complete BUSCOs (11) (v 5.4.6; analysis was performed on the proteins using the Galaxy website usegalaxy.org (12).

We compared our sequence to the *K. heterogenica* type strain NRRL-Y27499, as follows: the Illumina reads of the type strain (NCBI accession: SRR25935784) were mapped to the *K. heterogenica var. weizmannii* genome using BWA-mem (0.7.13) (13), and variant calling was performed with GATK (4.4.0.0) (14), SAMtools (1.9) (15), and Picard (2.22.8) (16). 96.34% of the reads mapped to the genome of our isolate. There were 18,779 SNPs in the entire genome, for a 0.13% difference. In addition, there were 7,506 indels, though most were short and in repetitive DNA elements. Taken together with virtually no differences in the rDNA locus (only one difference in 18S and two differences in ITS2 out of the entire locus), we believe that our isolate is *Kazachstania heterogenica* and should be known as the *weizmannii* strain.

## Data Availability

The sequences in this paper have been deposited in NCBI BioProject PRJNA936566, with the BioSample SAMN33355704. The NCBI Genome accession is GCA_036370985.1. This Whole-Genome Shotgun project has been deposited at DDBJ/ENA/GenBank under the accession JARZAD000000000. The version described in this paper is version JARZAD010000000. The fungus was deposited in the ATCC with the accession number PTA-127318.

## References

[1] Kurtzman CP, Robnett CJ, Ward JM, Brayton C, Gorelick P, Walsh TJ. 2005. Multigene phylogenetic analysis of pathogenic Candida species in the Kazachstania (Arxiozyma) telluris complex and description of their ascosporic states as Kazachstania bovina sp. nov., K. heterogenica sp. nov., K. pintolopesii sp. nov., and K. slooffiae. J Clin Microbiol 43:101–111. doi:10.1128/JCM.43.1.101-111.2005 15634957 PMC540161

[2] Kralova JS, Donic C, Dassa B, Livyatan I, Jansen PM, Ben-Dor S, Fidel L, Trzebanski S, Narunsky-Haziza L, Asraf O, Brenner O, Dafni H, Jona G, Boura-Halfon S, Stettner N, Segal E, Brunke S, Pilpel Y, Straussman R, Zeevi D, Bacher P, Hube B, Shlezinger N, Jung S. 2024. Competitive fungal commensalism mitigates candidiasis pathology. J Exp Med. doi:10.1101/2024.01.12.575358 PMC10949073 38497819

[3] Bcl2Fastq conversion software. https://support.illumina.com/sequencing/sequencing_software/bcl2fastq-conversion-software.html.

[4] Wick RR, Judd LM, Gorrie CL, Holt KE. 2017. Completing bacterial genome assemblies with multiplex MinION sequencing. Microb Genom 3:e000132. doi:10.1099/mgen.0.000132 29177090 PMC5695209

[5] Kolmogorov M, Yuan J, Lin Y, Pevzner PA. 2019. Assembly of long, error-prone reads using repeat graphs. Nat Biotechnol 37:540–546. doi:10.1038/s41587-019-0072-8 30936562

[6] Walker BJ, Abeel T, Shea T, Priest M, Abouelliel A, Sakthikumar S, Cuomo CA, Zeng Q, Wortman J, Young SK, Earl AM. 2014. Pilon: an integrated tool for comprehensive microbial variant detection and genome assembly improvement. PLoS One 9:e112963. doi:10.1371/journal.pone.0112963 25409509 PMC4237348

[7] Gurevich A, Saveliev V, Vyahhi N, Tesler G. 2013. QUAST: quality assessment tool for genome assemblies. Bioinformatics 29:1072–1075. doi:10.1093/bioinformatics/btt086 23422339 PMC3624806

[8] Proux-Wéra E, Armisén D, Byrne KP, Wolfe KH. 2012. A pipeline for automated annotation of yeast genome sequences by a conserved-synteny approach. BMC Bioinformatics 13:1–12. doi:10.1186/1471-2105-13-237 22984983 PMC3507789

[9] Steinbiss S, Silva-Franco F, Brunk B, Foth B, Hertz-Fowler C, Berriman M, Otto TD. 2016. Companion: a web server for annotation and analysis of parasite genomes. Nucleic Acids Res 44:W29–W34. doi:10.1093/nar/gkw292 27105845 PMC4987884

[10] Fu L, Niu B, Zhu Z, Wu S, Li W. 2012. CD-HIT: accelerated for clustering the next-generation sequencing data. Bioinformatics 28:3150–3152. doi:10.1093/bioinformatics/bts565 23060610 PMC3516142

[11] Simão FA, Waterhouse RM, Ioannidis P, Kriventseva EV, Zdobnov EM. 2015. BUSCO: assessing genome assembly and annotation completeness with single-copy orthologs. Bioinformatics 31:3210–3212. doi:10.1093/bioinformatics/btv351 26059717

[12] Afgan E, Nekrutenko A, Grüning BA, Blankenberg D, Goecks J, Schatz MC, Ostrovsky AE, Mahmoud A, Lonie AJ, Syme A, et al.. 2022. The galaxy platform for accessible, reproducible and collaborative biomedical analyses: 2022 update. Nucleic Acids Res 50:W345–W351. doi:10.1093/nar/gkac247 35446428 PMC9252830

[13] Li H. 2013. Aligning sequence reads, clone sequences and assembly Contigs with BWA-MEM. doi:10.48550/arXiv.1303.3997

[14] Van der AG, O’Connor B, Safari an OMC. 2020. Genomics in the cloud: using Docker, GATK, and WDL in Terra. In Genomics in the cloud 300

[15] Danecek P, Bonfield JK, Liddle J, Marshall J, Ohan V, Pollard MO, Whitwham A, Keane T, McCarthy SA, Davies RM, Li H. 2021. Twelve years of SAMtools and BCFtools. Gigascience 10:1–4. doi:10.1093/gigascience/giab008 PMC7931819 33590861

[16] Picard tools - by broad Institute. https://broadinstitute.github.io/picard.

